# Patient-derived glioblastoma stem cells respond differentially to targeted therapies

**DOI:** 10.18632/oncotarget.13415

**Published:** 2016-11-17

**Authors:** Pratik Kanabur, Sujuan Guo, Cara M. Rodgers, Gary R. Simonds, Deborah F. Kelly, Robert G. Gourdie, Scott S. Verbridge

**Affiliations:** ^1^ Virginia Tech Carilion Research Institute, Roanoke, VA 24016, United States; ^2^ Department of Biological Sciences and Pathobiology, Virginia-Maryland College of Veterinary Medicine, Virginia Tech, Blacksburg, VA 24061, United States; ^3^ Virginia Tech Carilion School of Medicine, Roanoke, VA 24016, United States; ^4^ Faculty of Health Science, Virginia Tech, Blacksburg, VA 24061, United States; ^5^ Virginia Tech-Wake Forest University School of Biomedical Engineering and Sciences, Blacksburg, VA 24061, United States; ^6^ Department of Emergency Medicine, Virginia Tech Carilion School of Medicine, Roanoke, VA 24016, United States; ^7^ Department of Neurosurgery, Carilion Clinic, Roanoke, VA 24016, United States; ^8^ Department of Biological Sciences, Virginia Tech, Blacksburg, VA 24061, United States

**Keywords:** glioblastoma, glioblastoma stem cells, patient-derived glioblastoma stem cells, targeted therapies

## Abstract

The dismal prognosis of glioblastoma is, at least in part, attributable to the difficulty in eradicating glioblastoma stem cells (GSCs). However, whether this difficulty is caused by the differential responses of GSCs to drugs remains to be determined. To address this, we isolated and characterized ten GSC lines from established cell lines, xenografts, or patient specimens. Six lines formed spheres in a regular culture condition, whereas the remaining four lines grew as monolayer. These adherent lines formed spheres only in plates coated with poly-2-hydroxyethyl methacrylate. The self-renewal capabilities of GSCs varied, with the cell density needed for sphere formation ranging from 4 to 23.8 cells/well. Moreover, a single non-adherent GSC either remained quiescent or divided into two cells in four-seven days. The stem cell identity of GSCs was further verified by the expression of nestin or glial fibrillary acidic protein. Of the two GSC lines that were injected in immunodeficient mice, only one line formed a tumor in two months. The protein levels of NOTCH1 and platelet derived growth factor receptor alpha positively correlated with the responsiveness of GSCs to γ-secretase inhibitor IX or imatinib, two compounds that inhibit these two proteins, respectively. Furthermore, a combination of temozolomide and a connexin 43 inhibitor robustly inhibited the growth of GSCs. Collectively, our results demonstrate that patient-derived GSCs exhibit different growth rates in culture, possess differential capabilities to form a tumor, and have varied responses to targeted therapies. Our findings underscore the importance of patient-derived GSCs in glioblastoma research and therapeutic development.

## INTRODUCTION

Glioblastoma (GBM) is the most common malignancy in the central nervous system and accounts for more than 45% of all malignant brain tumors [1]. The prognosis for patients with GBM is dismal, with a median survival time of 14.6 months following aggressive treatments (i.e. safe surgical removal, ionizing radiation, and chemotherapy) [2, 3]. After treatment, however, most patients have tumor recurrence and die within 5–7 months [4]. The percentage of GBM patients with a 5-year survival is approximately 5%, ranking it the most lethal among brain cancers [1]. Despite years of research into GBM's pathobiology and continuing years of clinical trials, there has only been a 2.5-month increase in survival [2], highlighting an urgent need for more efficacious treatments.

One important obstacle against effective treatment is the intra and inter-tumoral heterogeneity within GBMs, which is predominantly caused by the presence of self-renewing GBM stem cells (GSCs) [5–8]. GSCs are tumor cells with a neural stem cell-like phenotype that perhaps sustain tumor growth through asymmetric division and increase the tumor infiltration into neighboring tissues. In addition to tumor microenvironment and a poor drug penetration through the blood-brain barrier into the tumor, GSCs are another contributing factor to therapeutic resistance because these cells are refractory to radiation and chemo drugs [9, 10]. Also, the aggressive invasion of GBM cells, including GSCs, into the surrounding normal brain, often in non-expendable parts of the cranium, makes complete resection impossible and significantly increases resistance to the standard therapy regimen [3, 11, 12]. Molecular examination of GSCs has revealed defective apoptotic regulation, enhanced pro-survival signaling, and a strong propensity for tumor formation which together aids in the adaptation to environmental stress and resistance to treatment and also virtually assures tumor recurrence [13, 14]. Together, intra- and inter-tumor heterogeneity of GSCs may contribute to the multifaceted resistance and differential responses to cancer agents, and promote disease progression. Hence, it is critical to measure therapeutic effects among GSCs from individual tumors.

While prospectively distinguishing GSCs, which reside at the apex of tumor hierarchies, from their differentiated progeny remains challenging, the similarity of GSC to its normal counterpart neural stem cell has greatly helped identification and isolation of GSCs from bulk tumors [15–17]. Of note, it is still debated as to whether a cancer stem cell (CSC) is the cell origin of cancer; however, eliminating CSCs has become a very attractive therapeutic option to treat cancer and prevent cancer recurrence [18–23]. In general, the CSCs are able to self-renew, maintain sustained proliferation, and initiate tumor formation. Other disputed characteristics include certain numeric frequencies within a tumor, stem cell marker expression, the ability to differentiate into multiple cell lineages, and the propensity to form tumors. Hence, it is imperative to characterize these stem cells and determine if different cancer agents should be used to target these cells.

Many targeted therapies have been developed to eliminate GSCs. Among these therapeutic targets, NOTCH1 [24–31] and PDGFR [32–37] (platelet-derived growth factor receptors) have received more attention recently given their important roles in GSCs. Our previous research demonstrates that NOTCH1-positive GSCs are more potent to self renew than NOTCH1-negative cells, suggesting that NOTCH1-positive GSCs are more tumorigenic [31]. Blocking NOTCH1 has been applied to GSCs and this treatment has received promising therapeutic efficacy *in vitro* [24, 29, 30]. GSCs also highly express PDGFR, and targeting this gene using a multikinase inhibitor, imatinib, effectively kills GSCs [33, 36, 38–40]. We have recently found that a gap junction protein connexin 43 (Cx43) is highly enriched in GSCs and responsible for temozolomide (TMZ) resistance [41]. TMZ is a front-line chemo agent for GBM due to its excellent penetration through the blood-brain barrier [3]. This agent alkylates O6 guanine and causes DNA damage through the DNA mismatch repair pathway, thereby inducing cell death; however, GBM cells, including GSCs, develop a resistance to this drug [42, 43]. By using a new Cx43 inhibitor αCT1, the sensitivity of GSCs to TMZ has been significantly increased [41]. Nonetheless, whether individual GSCs have discrete responses to the drugs described above remains to be determined.

To target individual GSCs, we isolated 10 GSC lines from established cell lines, xenografts, and freshly dissected tumor tissues. We then characterized these lines and verified their stem cell identity and tumorigenicity. Furthermore, we measured the effect of different cancer drugs on these cells. Our results will facilitate the development of new and effective therapies to eradicate GSCs.

## RESULTS

### Isolation and characterization of individual GSCs

**Figure 1:**
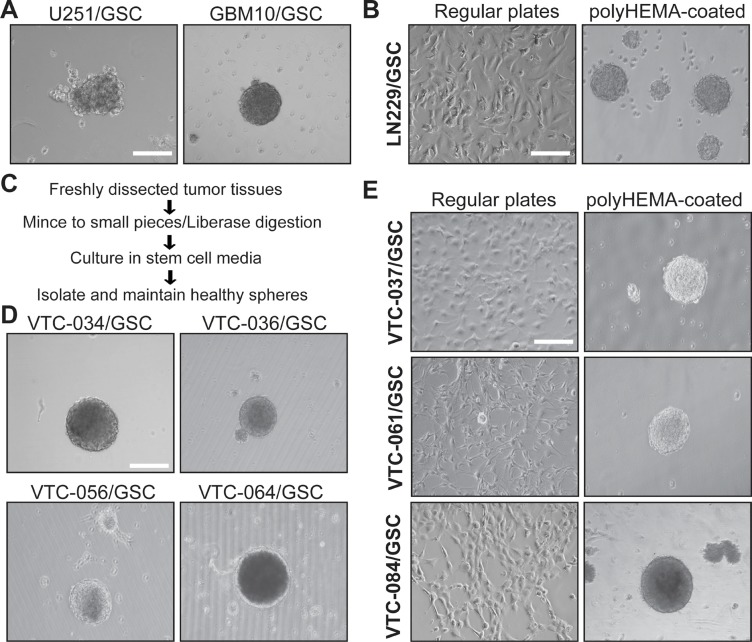
GSCs cultured in plates with or without polyHEMA coating (**A**) Spheres of U251/GSC and GBM10/GSC. GSCs were maintained in stem cell media as spheres. Pictures were taken using a Zeiss inverted microscope with a 10X lens. (**B**) LN229/GSCs. Cells were grown as monolayer (left panel) in stem cell media or as spheres (right panel) in poly-HEMA-coated plates. (**C**) Schematic diagram illustrating the steps of GSC isolation from freshly dissected tumor tissues. (**D**) Spheres of GSCs derived from patient tissues. (**E**) Patient-derived GSCs cultured in plates with or without polyHEMA coating. These GSCs were similar to LNC229/GSCs and highlighted in bold. Scale bar is 50 μm.

GSCs have been previously isolated from established GBM cell lines as well as primary tumor tissues using sphere formation or CD133-based cell sorting [15–17, 44–47]. To establish GSC lines, we first isolate GSCs from GBM cell lines U251 and LN229 and a xenograft line GBM10 using sphere formation. The serum-free stem cell medium contains B-27 supplement, fibroblast growth factor-2, and epidermal growth factor. This medium supports the growth of GSCs only. Differentiated tumor cells tend to die due to the lack of serum [46]. We found that U251 and GBM10 cells formed spheres after a month incubation in stem cell media (Figure 1A). However, LN229 cells grew as adherent monolayer (Figure 1B, left panel). After incubation in plates coated with polyHEMA [Poly (2-hydroxyethyl methacrylate] to prevent cell attachment, LN229/GSC formed spheres (Figure 1B, right panel). These results were consistent with previous work [41, 44, 46]. We then used the same approach to isolate GSCs from GBM tissues (outlined in Figure 1C). These tumor specimens were freshly obtained from the Neurosurgery Department at the Carilion Clinic (Roanoke, VA). We have successfully established seven primary GSC lines. VTC-034/GSC, VTC-036/GSC, VTC-056/GSC, and VTC-064/GSC grew as spheres (Figure 1D), whereas VTC-037/GSC, VTC-061/GSC, and VTC-084/GSC shared similar phenotypes with LN229/GSC (Figure 1E).

**Figure 2:**
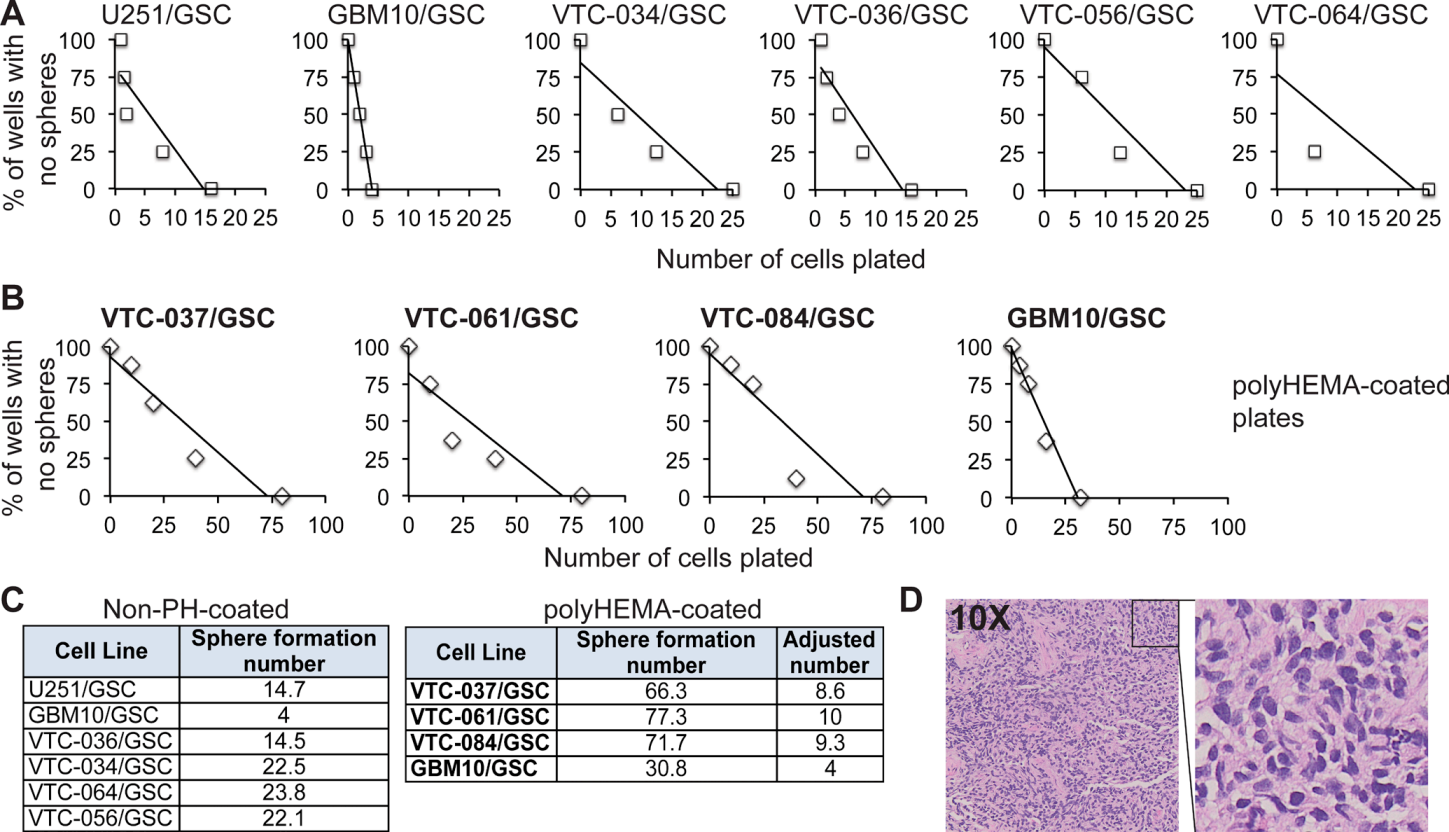
The propensity of GSCs to self-renew *in vitro* or to form a tumor *in vivo* (**A**) Sphere formation of GSCs. Single GSCs at different cell densities were plated. The percentages of wells with no spheres were plotted against the numbers of cells plated. A linear regression model was applied. (**B**) Sphere formation of GSCs in polyHEMA-coated plates. (**C**) Sphere formation numbers. The numbers that are required for sphere formation (abbreviated as sphere formation number) were determined based on the linear regression models (A and B). GBM10/GSCs were tested in plates with or without polyHEMA coating. The sphere formation numbers of patient-derived GSC lines (highlighted in bold) in polyHEMA-coated plates were adjusted based on the results from GBM10/GSCs. (**D**) Hematoxylin & Eosin staining of the subcutaneous tumor from VTC-036/GSCs.

To further characterize GSCs, we monitored the self-renewal of these lines. Self-renewal is determined by the capacity of GSCs to copy themselves [22]. We used the well-established sphere formation assay to measure the self-renewal of stem cells [15–17, 31]. The formation of spheres, which are clusters of undifferentiated cells, indicates active proliferation. However, spheres will only form when cells are placed at an appropriate density. We therefore used this assay to determine the minimal number of cells required for sphere formation. After single cells were plated at different densities, the number of wells with spheres per each density was counted. The percentages of wells with no spheres were plotted against the numbers of cells initially plated. A linear regression model was then applied to determine the number of cells needed for sphere formation (abbreviated as sphere formation number). The x-intercept of the linear model is the minimum required density to form a sphere. While the ideal stem cell can self-renew by itself to form a sphere, most cells need chemotactic factors from other cells to proliferate. Thus, the smaller the number is, the stronger capability is of the GSCs to self-renew. We found that 10 GSC lines proliferated at different rates (Figure 2A–2B). GBM10/GSCs had the lowest sphere formation number among all lines (Figure 2A and Figure 2C, left panel); however, the capability of GBM10/GSCs to form spheres significantly reduced in polyHEMA coated plates (Figure 2B and 2C, right panel). These results suggest that polyHEMA, while facilitating the formation of spheres, has a negative effect on GSC proliferation. In order to compare all patient-derived GSC lines together, we adjusted the sphere formation numbers of VTC-037/GSC, VTC-061/GSC, or VTC-084/GSC lines based on the results from GBM10/GSCs (Figure 2C, right panel). Among all patient-derived GSCs, the sphere formation numbers varied from 8.6 to 23.8, confirming the inter-tumor heterogeneity in individual GSCs. To test tumorigenicity, we implanted VTC-036/GSCs or VTC-037/GSCs in the flank of immunodeficient mice. A tumor with a diameter of 1 cm formed in 68 days in the mouse receiving VTC-036/GSCs. But there was no tumor formation in the mouse injected with VTC-037/GSCs. Hematoxylin and Eosin staining verified the identity of tumor cells (Figure 2D). Together these results indicate that GSCs from individual tumors often have different propensities to proliferate *in vitro* and to form tumors *in vivo*.

**Figure 3:**
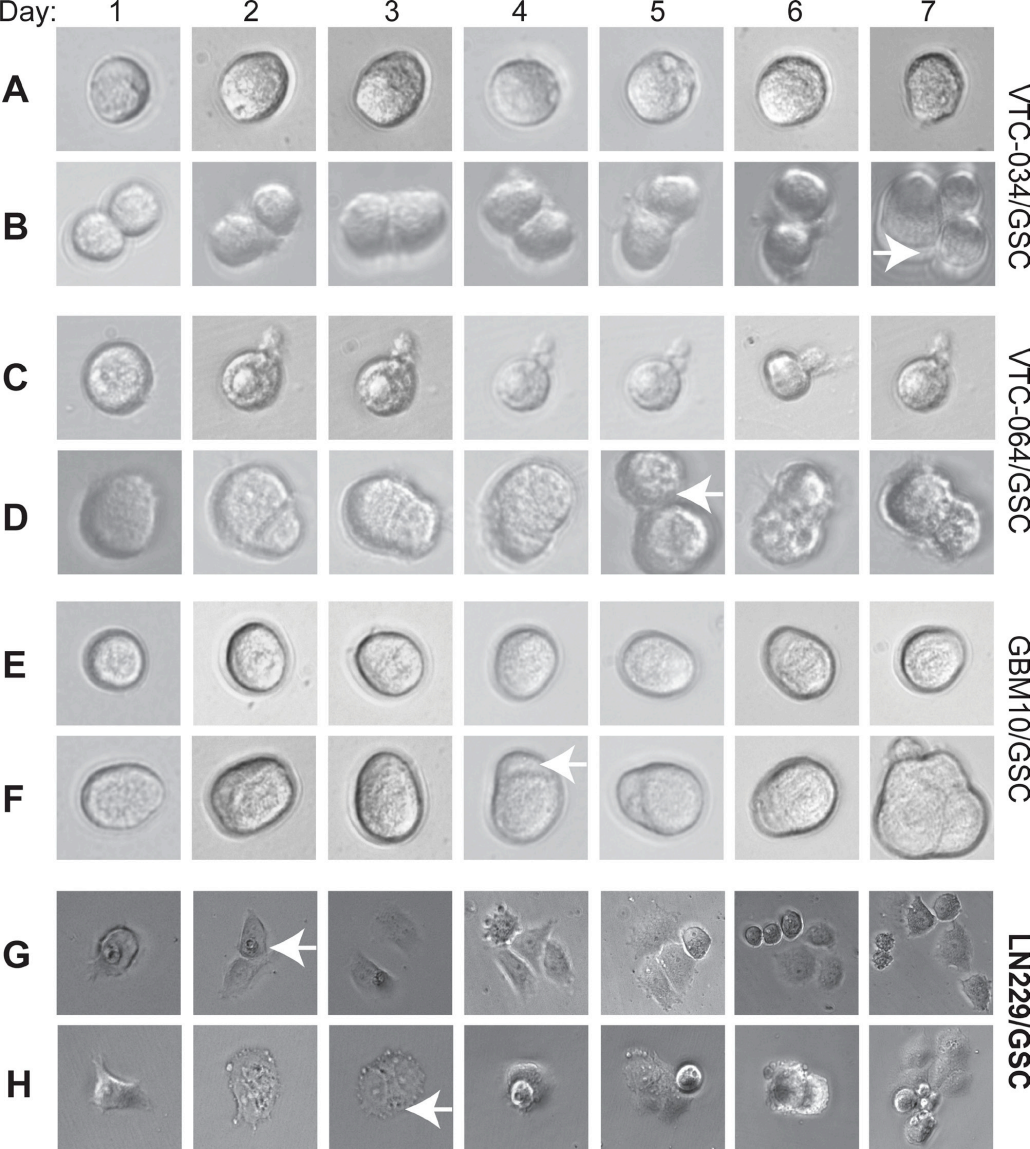
Dividing of single GSCs Single VTC-034/GSCs (**A–B**), VTC-064/GSCs (**C–D**), GBM10/GSCs (**E–F**), adherent LN229/GSCs (**G–H**) were plated and imaged using a 40X lens of an inverted microscope every day for seven consecutive days. Cropped images are shown. Images of adherent LN229/GSCs were in a different scale in order to show more cells. White arrows indicate the two-cell stages.

GSCs, similar to normal stem cells, tend to divide slowly and often keep quiescent. We then monitored the growth of single cells from three non-adherent GSC lines VTC-034/GSC, VTC-064/GSC, and GBM10/GSC. We found that some single GSCs remained at one-cell stage for 7 days, suggesting that they were quiescent (Figure 3A, 3B, and 3E). The times for a single VTC-034/GSC, VTC-064/GSC, or GBM10/GSC to divide into two cells were 7, 5, and 4 days, respectively (Figure 3B, 3D, and 3F, white arrows). Single adherent LN229/GSCs divided into two cells in 2–3 days and multi-cells in 4–5 days (Figure 3G and 3H). Hence, adherent LN229/GSCs grew much faster than non-adherent GSCs. Our results verify that patient-derived/non-adherent GSCs divide slowly.

**Figure 4:**
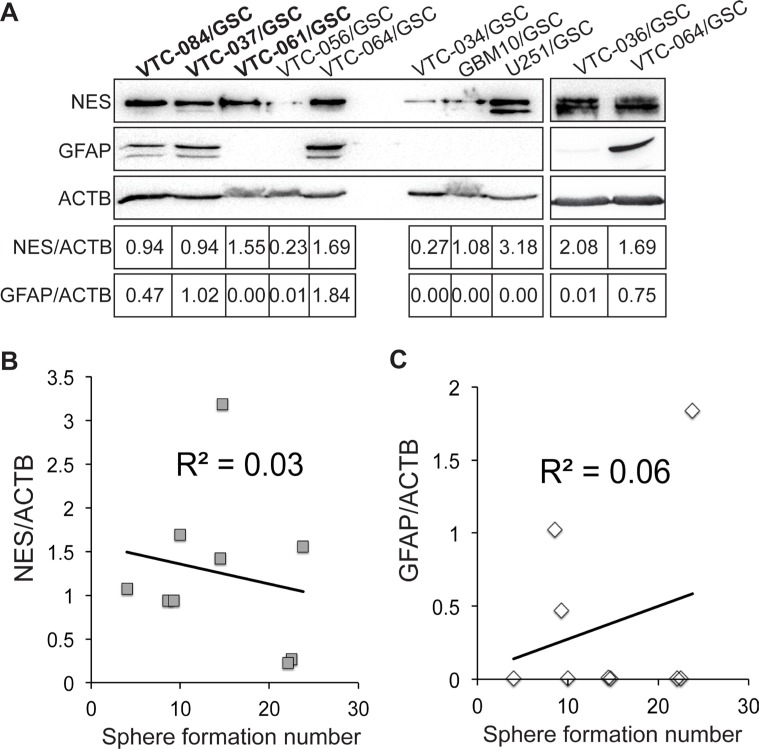
Expression of nestin and GFAP in GSCs (**A**) The protein levels of nestin (NES) and GFAP. β-actin (ACTB) was used as the loading control. Band intensities were quantified using Image J software. The ratios of NES/ACTB are shown. Correlations between sphere formation numbers and the protein levels of nestin (**B**) or GFAP (A–**C**) were determined using the linear regression model. Coefficients of determination (R^2^) are shown.

We next sought to determine the expression levels of a neural stem cell marker nestin (NES) and an astrocyte marker glial fibrillary acidic protein (GFAP) in GSCs using immunoblotting. We found that NES was expressed in GSC lines at different levels (Figure 4A). The relative band intensities (NES/ACTB) were from 0.23 to 3.18. In contrast, GFAP were only found in VTC-037/GSCs, VTC-064/GSCs, and VTC-084/GSCs, two of which formed spheres in polyHEMA-caoted plates only. The expression of GFAP in VTC-064/GSCs may be explained by the spontaneous differentiation of this line. In VTC-037/GSCs and VTC-084/GSCs, ployHEMA may contribute significantly to the differentiation and, thereby, inhibits the self-renewal (Figure 2C). In addition, the high levels of GFAP in VTC-037/GSCs may explain the failure of this line in tumor formation in mice. Unlike the aforementioned two GSC lines, VTC-061/GSCs did not express GFAP (Figure 4A), but formed spheres only in polyHEMA-coated plates (Figure 1E). Further linear regression analyses found no correlations between GSCs’ self-renewal and the protein levels of NES or GFAP (Figure 4B–4C). The coefficients of determination (R^2^) were 0.03 or 0.06, respectively. Hence, the expression levels of marker proteins in GSC lines derived from GBM patients can only be used to verify their stem cell identity, but not the propensity to proliferate. While PolyHEMA helps establishment of spheres, this agent affects stem cell growth, differentiation, and perhaps tumor formation. Cautions should be taken to interpret data from adherent GSCs cultured in polyHEMA-coated plates.

### Therapeutically targeting GSCs

**Figure 5:**
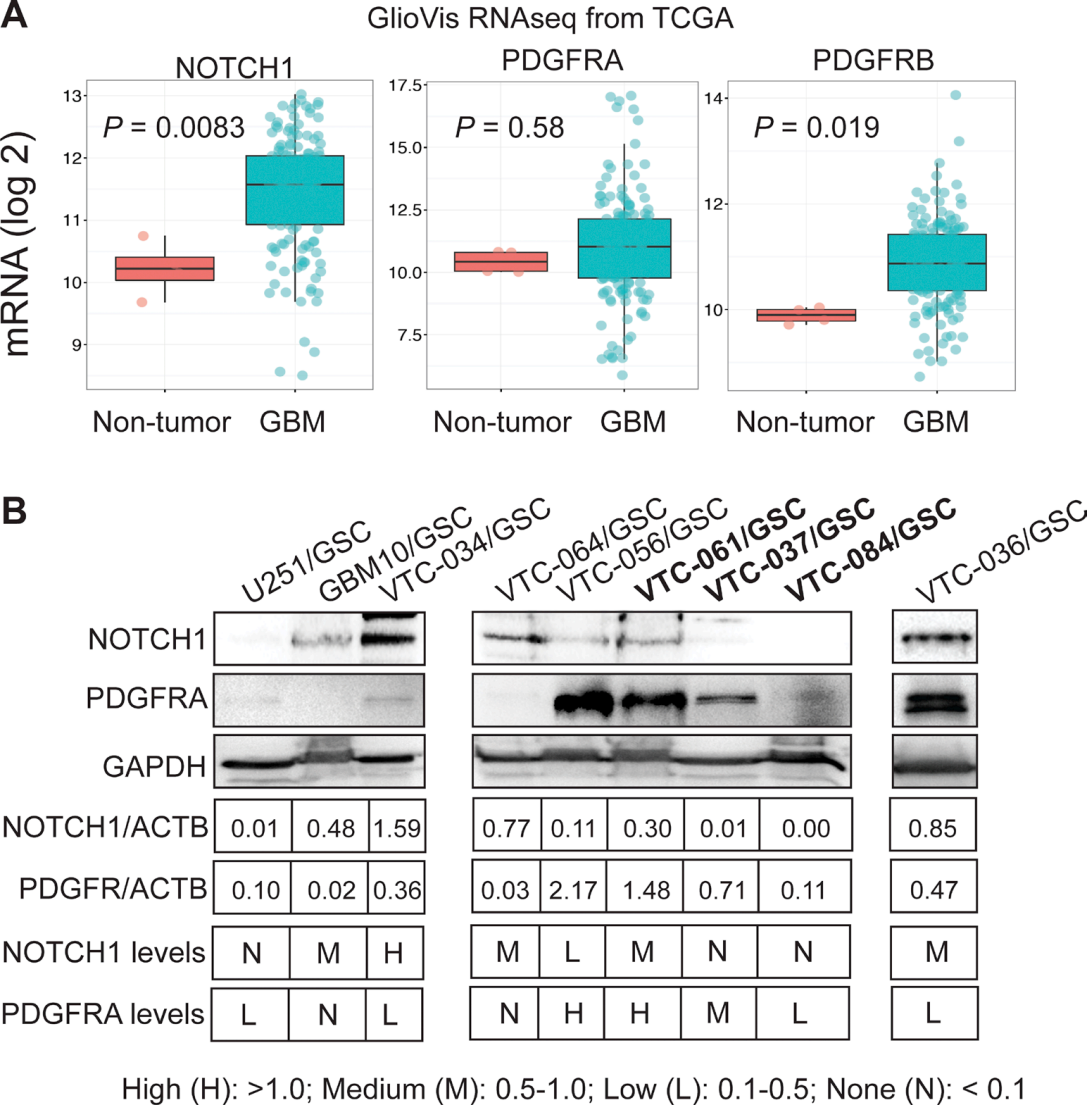
Expression of NOTCH1 and PDGFR in GBMs and GSCs (**A**) mRNA levels of NOTCH1, PDGFRA, and PDGFRB in GBMs. Gene expression data (RNAseq) were retrieved from the TCGA database and analyzed using the GlioVis program. The mRNA levels were compared between the non-tumor control and GBM samples. (**B**) Protein levels of NOTCH1 and PDGFR in GSCs determined by immunoblotting. Band intensities were measured using Image J software. The legend for categorizing protein levels is shown.

PDGFR and NOTCH1 are highly enriched in GBM, and targeting these two genes to treat GBM is currently under investigation [24–37]. By using the GlioVis program, we analyzed the mRNA levels of NOTCH1 gene and PDGFR isoforms (PDGFRA and PDGFRB) in more than 500 GBM patients from The Cancer Genome Atlas (TCGA) database. The levels of NOTCH1 and PDGFRB in GBM were significantly higher than those in non-tumor controls (*P* values were 0.0085 or 0.019, respectively), whereas PDGFRA levels were similar (*P value* was 0.58; Figure 5A), consistent with previous reports [24–37]. However, whether these two genes are differentially expressed among individual tumors are not clear. We thus measured the protein levels of NOTCH1 and PDGFRA in GSCs. We found that NOTCH1 and PDGFRA were reciprocally expressed in some GSC lines (Figure 5B). For example, U251/GSC, VTC-037/GSC, and VTC-084/GSC lines were NOTCH1-negative, but expressed PDGFRA at low or medium levels. In contrast, GBM10/GSCs and VTC-061/GSCs expressed NOTCH1 at medium levels, but PDGFRA was not detected in these cells. The remaining GSC lines expressed both proteins. Hence, NOTCH1 and PDGFRA are differentially expressed in GSCs isolated from individual GBM tumors.

**Figure 6:**
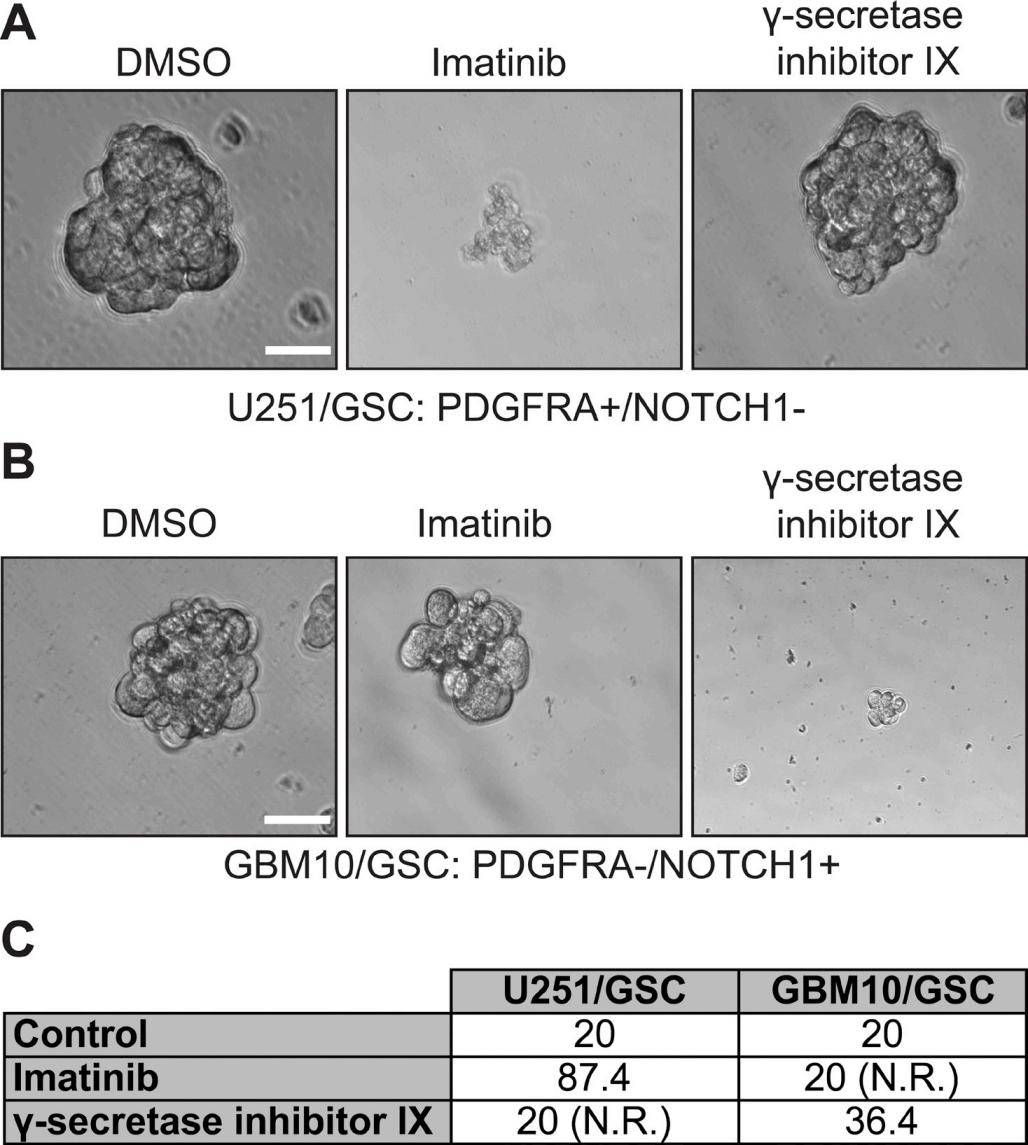
The effect of imatinib and γ-secretase inhibitor IX on U251/GSCs and GBM10/GSCs U251/GSCs and GBM10/GSCs were plated at different cell densities and treated with vehicle DMSO, imatinib (10 μM), or γ-secretase inhibitor IX (20 μM). The cells were imaged (**A** and **B**) using a 40X lens of an inverted microscope. The sphere formation numbers were calculated as described in Methods section (**C**). Scale bar is 10 μm.

**Figure 7:**
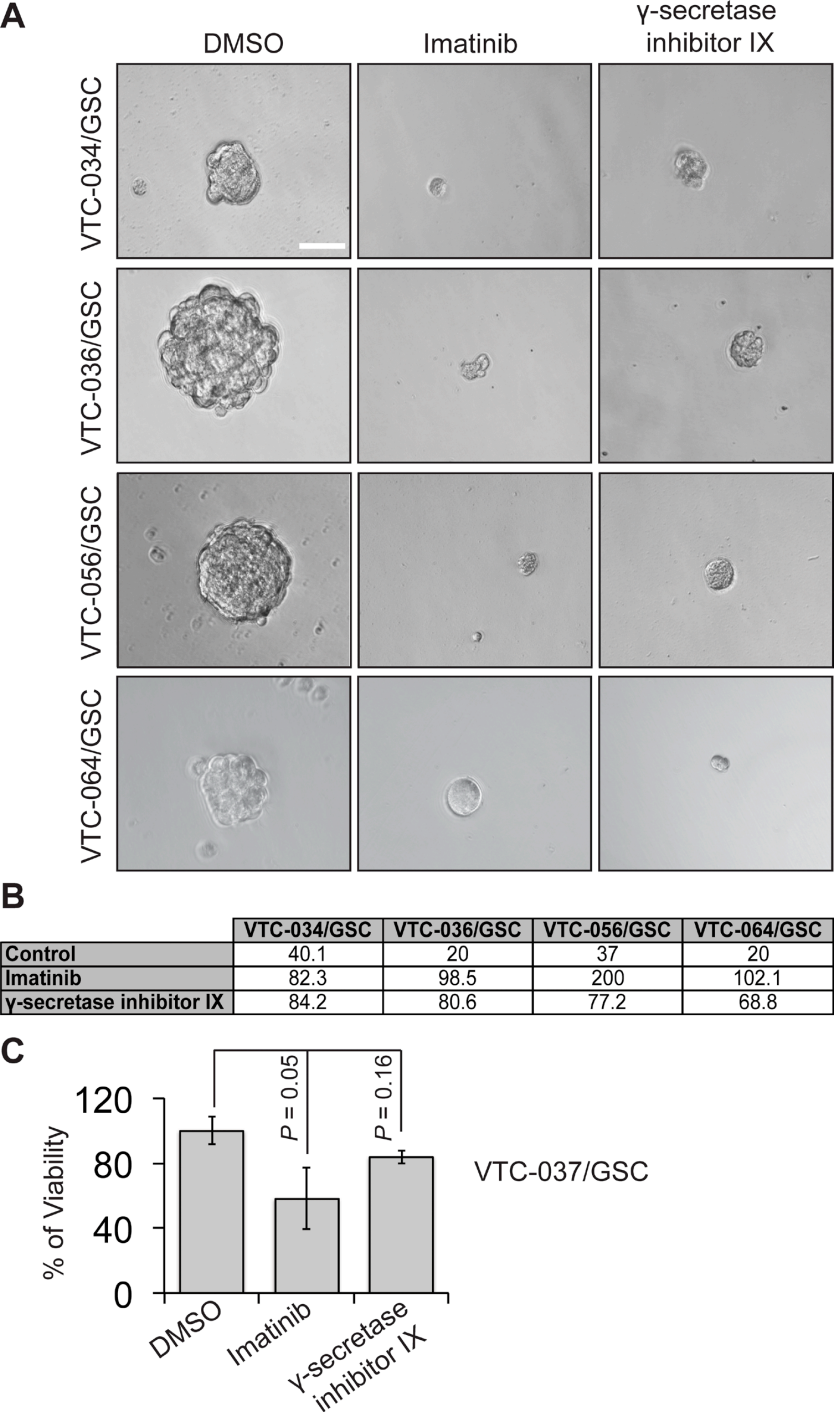
The effect of imatinib and γ-secretase inhibitor IX on patient-derived GSCs GSCs were plated at different cell densities and treated with vehicle DMSO, imatinib (10 μM), or γ-secretase inhibitor IX (20 μM). Cells were imaged using a 40 X lens of an inverted microscope (**A**). The sphere formation numbers were determined based on numbers of cells plated and the percentages of wells with no spheres (**B**). The responses of VTC-037/GSC to these drugs were determined using the MTS viability assay (**C**). Scale bar is 10 μm. Error bars represent standard deviations from three independent experiments.

We next sought to determine whether different levels of NOTCH1 and PDGFRA in GSCs yield discrete responses to drugs targeting these two proteins. To test this, we used γ-secretase inhibitor IX to block NOTCH1 signaling and a multikinase inhibitor imatinib to suppress PDGFRA activity. Both drugs have shown effective growth inhibition of GBM cells, including GSCs [24–37]. We first tested the self-renewal of U251/GSC (NOTCH1-negative/PDGFRA-positive) and GBM10/GSC (NOTCH1-positive/PDGFRA-negative) because these two lines reciprocally expressed PDGFRA and NOTCH1. As expected, the self-renewal of U251/GSCs was significantly reduced upon treatment of imatinib but not that of γ-secretase inhibitor IX (Figure 6A and 6C). In stark contrast, GBM10/GSCs only responded to γ-secretase inhibitor IX (Figure 6B–6C). In patient-derived GSC lines, which expressed both proteins, γ-secretase inhibitor IX and imatinib substantially inhibited the self-renewal of VTC-034/GSCs, VTC-036/GSCs, VTC-056/GSCs, and VTC064/GSCs (Figure 7A–7B). Because VTC-037/GSC grew adherently in stem cell media (Figure 1E), it was difficult to measure drug effects using the sphere formation assay. By using the MTS viability assay, we found that the viability of VTC-037/GSC (NOTCH1-negative/PDGFRA-positive) declined when cells were treated with imatinib (*P value* was 0.05). However, γ-secretase inhibitor IX had no effect on cell viability (*P* value was 0.16; Figure 7C), consistent with our previous results (Figure 6). Taken together, our results demonstrate that the protein levels of NOTCH1 or PDGFRA determine the effectiveness of γ-secretase inhibitor IX or imatinib in GSCs. It is therefore critical to measure the protein levels of clinical samples before applying targeted therapies to GBM patients.

**Figure 8:**
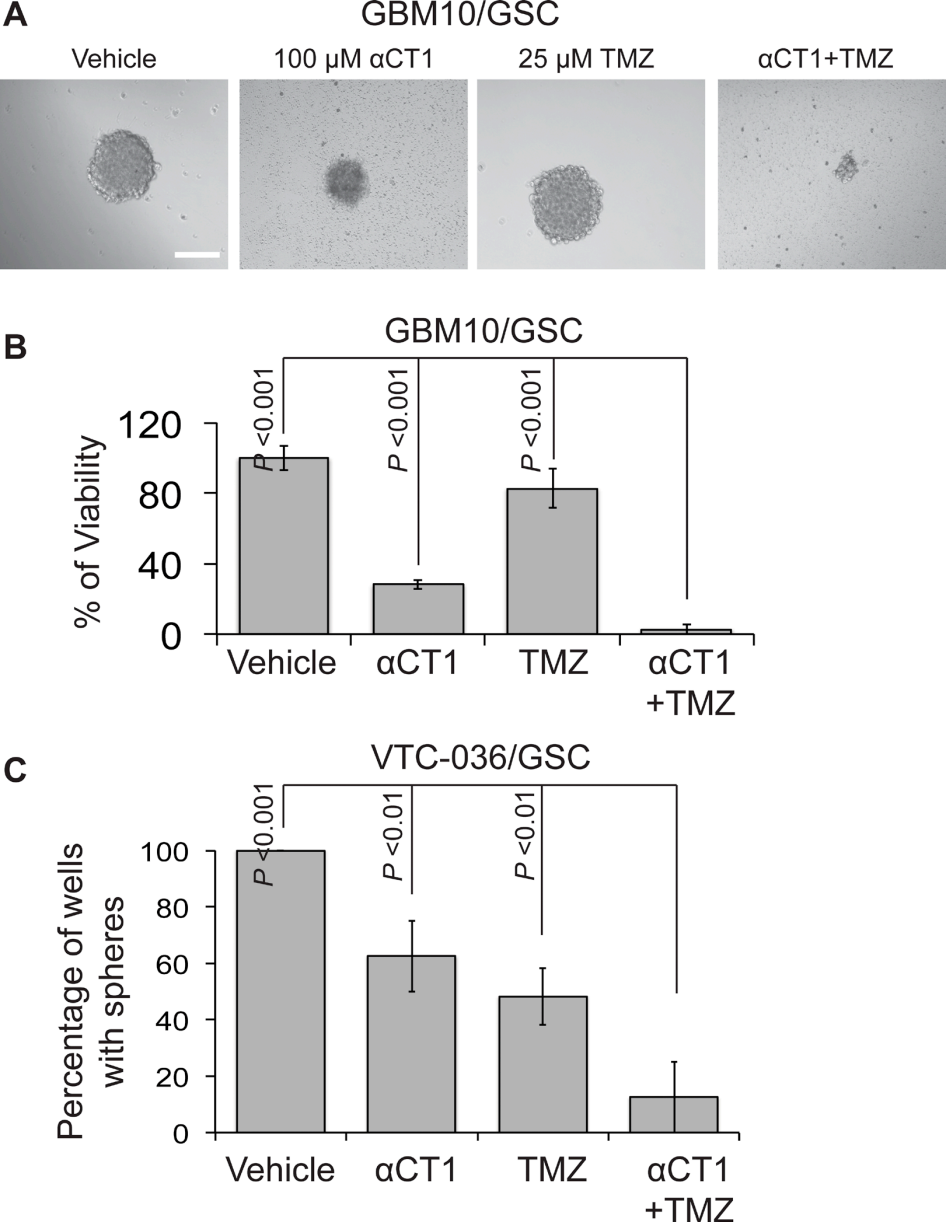
The effect of αCT1 and TMZ in GBM10/GSCs and VTC-036/GSCs (**A**) GBM10/GSCs treated with αCT1 and/or TMZ were imaged using a 40 X lens of an inverted microscope. Scale bar is 10 μm. (**B**) The viability of GBM10/GSCs determined by the MTS viability assay. (**C**) The sphere formation of VTC-036/GSCs. Error bars represent standard deviations from three independent experiments.

We have recently showed that Cx43 is pivotal for TMZ resistance and a Cx43 inhibitor αCT1 helps circumvent this resistance [41]. To further verify the therapeutic benefits of TMZ and αCT1 combinational treatment, we measured the viability of GBM10/GSC or the self-renewal of VTC-036/GSCs when these cells were treated with above drugs. αCT1 alone significantly inhibited the viability of GBM10/GSC (Figure 8A–8B) and the self-renewal of VTC-036/GSCs (Figure 8C). A combination of αCT1and TMZ further strengthened the inhibition of cell viability and self-renewal. Hence, αCT1 and TMZ can be used as a combinational treatment to eliminate GSCs.

## DISCUSSION

Patient derived xenograft (PDX) models are superior to xenograft models from established cells lines in capitulating the pathobiology of GBM, particularly tumor heterogeneity [48–51]. This is because established cell lines have homogeneous, undifferentiated histology and consequently, no longer retain the original molecular characteristics of parental tumors [52, 53]. PDX models, on the other hand, have high penetrance and short latency *in vivo*, but most importantly can predict clinical success faithfully and allow mechanistic studies of action by recapitulating the genomic diversity and histopathologic heterogeneity observed in patient tumors [48, 50]. These models have been shown, in addition, to replicate the patient response to therapies such as radiation and chemotherapy [48, 49, 52, 54]. Thus, using PDX models could allow for a personalized and precision medicine.

PDX models could be used for drug screening, which is supported by our finding that the expression levels of PDGFRA or NOTCH1 positively correlate with the susceptibility of individual GSCs to imatinib and γ-secretase inhibitor IX. Using this initial screening could decrease the large failure rates of drugs that reach phase III clinical trails [55]. In addition, PDX models are important for the development of biomarkers [50]. We have recently identified a set of kinase genes that exhibit a strong association with the diagnosis and prognosis of recurrent GBMs [56]. GSCs are hypothetically critical for the development of tumor recurrence. Hence, it is imperative to investigate these candidate kinases in the patient-derived GSCs reported herein and to explore their roles in GSCs and tumor formation/recurrence.

Based on the results described, we conclude that GSCs isolated from different individual tumors have discrete capabilities to grow in culture and that the expression levels of therapeutic targets in individual GSCs determine the effectiveness of corresponding targeted therapies. Our results demonstrate that the GSC lines we have established are suitable for testing the efficacy of cancer drugs, which will facilitate the discovery of GSC-eradicating drugs and provide cell models for the development of precision medicine for GBM patients.

## MATERIALS AND METHODS

### Cell Lines

Human GBM cell lines U251 and LN229 and primary cells isolated from GBM10 xenograft were maintained in Dulbecco's Modified Eagle Medium (DMEM, Life Technologies Corporation) supplemented with 10% fetal bovine serum (FBS; Atlas Biologicals, Inc.), streptomycin (100 μg/ml), and penicillin (100 IU/ml). Human GSC lines were maintained as spheres in stem cell media, which include DMEM, Gibco^®^ B-27^®^ Supplements (Life Technologies Corporation), 20 ng/ml FGF-2 (GenScript), and 20 ng/ml EGF (GenScript). LN229/GSCs, VTC-037/GSCs, VTC-061/GSCs, and VTC-084/GSCs were either grown as monolayer in above stem cell media or as spheres in flasks coated with poly(2-hydroxyethyl methacrylate) (PolyHEMA, Sigma-Aldrich Co. LLC).

### Isolation and preparation of primary GBM cells and GSCs

Institutional Review Board at the Carilion Clinic has approved the use of human GBM patient specimens. GSCs were isolated as described previously with modifications [15–17, 44–47]. Freshly resected human GBM tumors (pathologically confirmed) were minced into small pieces. Single cells were prepared using Liberase (Roche Diagnostics) according to manufacturer's instructions. Red blood cells were removed using the Red Blood Cell Lysis Solution (Miltenyi Biotec Inc). Isolated GSCs grew as spheres after 1 to 2 months of continuous culturing in stem cell media. During this course, dead cells were removed and healthy spheres were collected by low speed centrifugation (1000 rpm for 30 seconds). When spheres were visible by naked eyes, they were dissociated to single cells using TrypLE (Thermo-Fisher Scientific) and were cultured in stem cell media until visible spheres formed. Healthy spheres were frozen in stem cell media with 7% DMSO. Healthy spheres were imaged using a Zeiss inverted microscope.

### Sphere formation assay

Sphere-formation assays were performed as described earlier [31, 57]. Briefly, GSCs were inoculated in a 96-well plate at cell densities from 1 to 256 cells per well. Two to three weeks later, wells with or without spheres were counted. The percentage of wells with no spheres was defined as the ratio of wells without spheres to wells initially plated. A linear regression model (y = –*a*x+*b*) was applied to calculate the number of cells required for sphere formation (abbreviated as sphere formation number). The following formula was used: sphere formation number (x-intercept) = –*b*/*a*. The constants *a* and *b* were determined by the linear regression model.

In some experiments, GSCs selected for drug treatment were subject to a modified self-renewal assay. GSCs were plated in a 96 well plate at cell densities from 10 to 80 cells per well. GSCs were treated with DMSO, 10 μM of imatinib, or 20 μM of γ-secretase inhibitor IX. After 2 weeks the number of wells with spheres was counted and the sphere pictures were taken using an inverted microscope with a 40X lens. In one of the drug treatment experiments, GSCs were plated into a 96 well plate at a cell density of 50 cells per well. GSCs were treated with vehicle, 50 μM of TMZ, 100 μM of αCT1, or a combination of TMZ and αCT1 αCT1 treatment was repeated every fourth day for 2 doses.

### Single cell analysis

To analyze single GSCs, one GSC was plated in a well of a 96-well plate. The cell was cultured in stem cell media and imaged using a Zeiss inverted microscope every day for 7 consecutive days.

### Immunoblotting

Immunoblotting was performed as described in our previous reports [31, 41, 56, 58]. In brief, cells were lysed and total protein was quantified using the Bradford assay (Bio-Rad Laboratories Inc.). An equal amount of total protein (20–25 μg) in each sample was loaded onto an SDS-PAGE gel. After transferring to PVDF membrane, the blot was incubated with antibodies. Antibodies were diluted as follows: anti-Nestin (Santa Cruz Biotechnology, Inc., 1:200), anti-NOTCH1 (Cell Signaling Technology, 1:1000), anti-GFAP (Cell Signaling Technology, 1:1000), anti-PDGFRA (Cell Signaling Technology, 1:1000), anti-B-Actin (SigmaAldrich Co. LLC, 1:10000), and anti-GAPDH (Santa Cruz Biotechnology, Inc., 1:1000). Images were taken using a ChemiDoc MP System (Bio-Rad Laboratories Inc.).

### MTS Viability assays

MTS viability assays were described previously [41, 58]. In brief, 2.5 × 10^3^ GSCs were plated in a 96-well plate. Cells were then treated with DMSO, 50 μM of TMZ, 100 μM of αCT1, or a combination of TMZ and αCT1. αCT1 treatment was repeated every fourth day for 2 doses. In another set of experiments, cells were treated with imatinib or γ-secretase inhibitor IX as described above. After one week, cell viability was monitored using the MTS assay. 10 μl of MTS (Promega) was added to each well, then incubated at 37°C for 1 h. The absorbance at 490 nm was measured using a FilterMax F3 microplate reader (Molecular Devices, LLC) according to manufacturer's instructions. Percent cell viability was obtained by dividing the absorbance of treatment groups with those of untreated groups.

### Mouse experiments

Mouse experiments were performed based on methods described previously, with modifications [41, 58]. All animal studies were approved by the Institutional Animal Care and Use Committee (IACUC) of Virginia Tech. 10^5^ of VTC-036/GSCs or VTC-037/GSCs were subcutaneously injected into SCID/beige mice (Harlan, ENVIGO). A tumor from VTC-036/VTCs with a diameter of 1 cm formed in 68 days, whereas no tumor was found in the mouse receiving VTC-037/GSCs. The tumor was harvested and stained by Hematoxylin and Eosin.

### Statistical analyses

Student's *t* test and one-way ANNOVA were used to determine the difference among treatment groups.

## Abbreviations

CSC: cancer stem cell
GBM: glioblastoma
GFAP: glial fibrillary acidic protein
GSC: glioblastoma stem cell
NES: nestin
PDGFR: platelet-derived growth factor receptor
PDX: patient-derived xenograft
PolyHEMA: poly(2-hydroxyethyl methacrylate)
TMZ: temozolomide
